# Role of DPP-4 and SGLT2 Inhibitors Connected to Alzheimer Disease in Type 2 Diabetes Mellitus

**DOI:** 10.3389/fnins.2021.708547

**Published:** 2021-08-11

**Authors:** A Young Sim, Sumit Barua, Jong Youl Kim, Yong-ho Lee, Jong Eun Lee

**Affiliations:** ^1^Department of Anatomy, Yonsei University College of Medicine, Seoul, South Korea; ^2^Brain Korea 21 Plus Project for Medical Science, Yonsei University College of Medicine, Seoul, South Korea; ^3^Department of Internal Medicine, Yonsei University College of Medicine, Seoul, South Korea; ^4^Brain Research Institute, Yonsei University College of Medicine, Seoul, South Korea

**Keywords:** insulin signaling, insulin resistance, Alzheimer’s disease, type 2 diabetes mellitus, DPP-4 inhibitor, SGLT2 inhibitor

## Abstract

Alzheimer’s disease (AD) is characterized by memory loss and cognitive decline. Additionally, abnormal extracellular amyloid plaques accumulation and nerve damage caused by intracellular neurofibrillary tangles, and tau protein are characteristic of AD. Furthermore, AD is associated with oxidative stress, impaired mitochondrial structure and function, denormalization, and inflammatory responses. Recently, besides the amyloid β hypothesis, another hypothesis linking AD to systemic diseases has been put forth by multiple studies as a probable cause for AD. Particularly, type 2 diabetes mellitus (T2DM) and its features, including hyperinsulinemia, and chronic hyperglycemia with an inflammatory response, have been shown to be closely related to AD through insulin resistance. The brain cannot synthesize or store glucose, but it does require glucose, and the use of glucose in the brain is higher than that in any other organ in the mammalian body. One of the therapeutic drugs for T2DM, dipeptidyl peptidase-4 (DPP-4) inhibitor, suppresses the degradation of incretins, glucagon-like peptides and glucose-dependent insulinotropic peptide. Sodium-glucose cotransporter 2 (SGLT2) inhibitors, recently used in T2DM treatment, have a unique mechanism of action *via* inhibition of renal glucose reabsorption, and which is different from the mechanisms of previously used medications. This manuscript reviews the pathophysiological relationship between the two diseases, AD and T2DM, and the pharmacological effects of therapeutic T2DM drugs, especially DPP-4 inhibitors, and SGLT2 inhibitors.

## Introduction

Alzheimer’s disease (AD) and type 2 diabetes mellitus (T2DM) are two of the most common disorders affecting older adults. AD occurs in 60–80% of the elderly population as the most common neurocognitive disorder and type of dementia. Clinically, AD is characterized by progressive memory loss and decreased cognitive function, leading to premature death several years after diagnosis. The most common pathological features of AD are the abnormal accumulation of amyloid plaques due to the aggregation of amyloid β (Aβ) peptides and neurofibrillary tangles (NFT) consisting of hyperphosphorylated tau protein. Recent studies have revealed that AD is associated with extracellular amyloid plaques, intracellular NFT, neuronal loss, and cellular damage. It is caused by oxidative stress, abnormal mitochondrial structure and function, inflammation, and aging (Kandimalla et al., 2017). This injury has also been associated with conditions related to insulin resistance, including hyperinsulinemia, chronic hyperglycemia, inflammation, and vascular changes. It was also confirmed that approximately 80% of AD patients are affected by insulin resistance or T2DM (de la Monte, 2014). This allows the mechanical relationship between T2DM and AD to be better understood. Research results have shown that AD can be regarded as a metabolic disorder with impaired brain glucose uptake and energy production. Therefore, studies on the causes of AD, based on the potential neuroprotective effects of anti-diabetic drugs and their direct and indirect mechanisms of action, are ongoing (Boccardi et al., 2019). In this review, we provide a summary of the mechanisms that link AD and T2DM; thereafter, we focus on the principal drugs of T2DM and explore their potential as suitable candidates for the treatment of AD.

## Insulin as a Mediator of T2DM

The worldwide incidence of diabetes, which is a chronic metabolic disorder, is rapidly increasing. Diabetes can be classified as types 1 and 2. T2DM, which accounts for 95% of all cases of diabetes, is characterized by hyperinsulinemia and insulin resistance (Taylor, 2012). Another feature of T2DM is the formation of amyloid polypeptides, which induce pancreatic β cell dysfunction (Marzban et al., 2003). Insulin resistance and amyloid peptides reduce the absorption of blood glucose and ultimately induce chronic hyperglycemia, one of the pathological features of T2DM (Chatterjee and Mudher, 2018). Maintaining the insulin secretory function and ameliorating insulin resistance are important in the management of T2DM.

### Insulin Signaling

Insulin is a hormone secreted by the β cells of the pancreas in response to high glucose levels. The binding of insulin to the insulin receptor (IR) initiates insulin signaling.

During insulin binding, IR auto-phosphorylates tyrosine residues in the intracellular portion of the receptor and then rapidly phosphorylates the tyrosine residues of C substrates 1 to 4 (IRS1-4; Lavan et al., 1997). IRS converts insulin *via* several pathways, the most well-known of which is the phosphoinositide 3-kinase (PIK3)/protein kinase B (AKT)/mechanistic target of rapamycin (mTOR) pathway of IRS1. When the serine residues of IRS1 and IRS2 are phosphorylated, these get separated from the IR, tyrosine phosphorylation of IRS is reduced, and the downward regulation of insulin signal is inhibited (Mothe and Van Obberghen, 1996).

In addition, insulin maintains glucose homeostasis by suppressing glycogen synthase kinase-3 β (GSK3-β), which regulates glucose production and glucose consumption by muscle and adipose tissue after passing through the glucose transporter type 4 (GLUT4; Guo, 2014; Roberts et al., 2014).

### Insulin Resistance

Insulin resistance indicates a reduced function of insulin in target tissues, such as the liver, muscles, and adipose tissue. The ability of IRS to get activated and transmit downstream signals is diminished in insulin-responsive tissues, leading to impaired insulin secretion and insulin dysfunction, which are major causes of diabetes (Leng et al., 2004; Guo, 2014).

Insulin resistance in skeletal muscle reduces glucose intake, making it difficult to regulate muscle glycogen synthesis (Hunter and Garvey, 1998). This is considered to occur due to the suppression of *GLUT4* gene by excessive free fatty acids. High saturated fatty acid levels, which can suppress normal IRS1 tyrosine phosphorylation and induce insulin resistance in skeletal muscle, show a correlation with skeletal muscle insulin activity (Roden et al., 1996; Pan et al., 1997). Recent studies investigating the deformation of O-linked-β-N-acetylglucosamine (OGlcNAc) protein found that OGlcNAc transferase and β-anomalous variants of N-acetylglucosamine [mediated by O-GlcNAcase (OGA)] are IRS Ser/Thr residues. An important function of the liver is to produce and store glycogen in a glucose reservoir that is readily available to the body (Ma and Hart, 2013). Glucose production during normal postprandial state with glycogenolysis is sufficient to meet the energy needs of the brain and other body tissues. However, insulin resistance results in a systemic insulin resistance phenomenon (Beck-Nielsen et al., 2002; Bugianesi et al., 2005) which causes the body tissues to be deprived of glucose.

## Insulin in the Brain

Insulin receptors are expressed in all brain cells, but the differences in expression levels vary considerably from region to region and are most visible in the cerebral cortex, striatum, and cerebellum. This suggests that insulin signaling is important in the brain and plays various roles (Arnold et al., 2018). Insulin and insulin-like growth factor (IGF) signaling mechanisms in the brain are important for maintaining synaptic plasticity, and function (Boucher et al., 2014). When insulin binds to an IR, multiple tyrosine residues are auto-phosphorylated to activate IRS1 and IRS2, which mediate downstream signaling through PIK3. This PIK3 activates AKT and suppresses activity at serine 9 residues *via* GSK3-β phosphorylation, leading to glycogen synthesis (Avila et al., 2012). The PIK/AKT pathway stimulates excitatory and inhibitory cell membrane receptors to regulate synaptic plasticity, enhance N-Methyl-D-aspartic acid (NMDA) receptor-mediated long-term potentiation and neurotransmitter activity, and is important for learning and memory. Additionally, the PIK/AKT pathway increases cortical glucose metabolism (Farrar et al., 2005; Bradley et al., 2012). However, in an abnormal state, GSK3-β is overactivated and tau is phosphorylated; this hyperphosphorylated tau gets aggregated and entangled in nerve fibers (Avila et al., 2010). GSK3-β also acts as a mediator of cell death, increasing the production of Aβ (Qu et al., 2014; Meng et al., 2020).

Insulin activates the mitogen-activated protein kinase (MAPK) pathway, leading to Ras activation and activation of rapidly accelerated fibrosarcoma (Raf), MAPK/ERK kinase (MEK), and extracellular signal-regulated kinase (ERK) in the protoplasmic membrane (Zhang et al., 2011). Although the direct role of the MAPK pathway components that mediate AD pathology has not yet been clarified, recent studies have reported that ERK plays an important role in synaptogenesis, learning, and memory function and has neuroprotective functions (Thiels and Klann, 2001).

Epidemiological studies and neuroimaging studies of the brain have indicated that insulin and IGF signaling pathways are important for the preservation and maintenance of learning and memory processes, and it can be confirmed that the function of learning and memory is improved in AD patients with intranasal insulin injection (Benedict et al., 2007).

## AD Caused by Insulin Resistance in the Brain

Alzheimer’s disease can be classified into two clinical subtypes: familial AD (fAD) and sporadic AD (sAD). Although the two types of disease (fAD and sAD) exhibit similar pathological phenotypes such as presence of plaques, tangles, synaptic damage, and neuronal loss, the factors that induce the neurodegenerative process are completely different. Pathological accumulation in fAD occurs due to the presence of autosomal dominant mutations in one of the three genes: amyloid protein precursor (APP), presenilin-1, or presenilin-2 (Querfurth and LaFerla, 2010). However, the cause of sAD, which accounts for the majority of AD cases, is complex and multifactorial based on the combination of genetic factors, epigenetic factors, and lifestyle-related factors. Moreover, most sAD patients are elderly individuals with various comorbidities (e.g., stroke, stress, diabetes, seizures, osteoporosis, and kidney disease) that can significantly increase the complexity underlying the pathogenesis of sAD (Doraiswamy et al., 2002; Magaki et al., 2014; Aubert et al., 2015).

Many recent studies have confirmed that insulin signaling impaired due to insulin resistance also occurs in AD (Talbot et al., 2012). In fluorodeoxyglucose (FDG)–positron emission tomography (PET) studies of the brains of patients with “early-stage” AD, AD was referred to as “type 3 diabetes” because of reduced glucose intake (de la Monte and Wands, 2008).

The important role of insulin in the peripheral system is well known and has been widely studied, but studies on insulin function in the central nervous system are currently underway. Previously, it was believed that due to the size of insulin, insulin could not pass through the brain-blood barrier (BBB), and the brain was considered to be insulin-independent; however, some studies have shown that IRs are expressed in the brain, and there are several mechanisms to support the presence of insulin (Banks, 2004; Gray et al., 2014).

Recent studies have established that insulin is transported through the BBB *via* carrier-mediated, saturated, and temperature-sensitive active processes. All types of brain cells, including neurons, have insulin signaling pathways, and insulin regulates the concentration of neurotransmitters such as acetylcholine, recovery, differentiation, proliferation, regeneration, and neuronal cell death (de la Monte et al., 2003; Goberdhan and Wilson, 2003; Russo et al., 2005).

Studies have shown that ICR mice on a long-term high-fat diet (HFD) developed T2DM with insulin resistance in both the body and brain, along with Alzheimer’s pathologies such as cognitive deficits, Aβ accumulation, and hyperphosphorylated tau. Aβ oligomers remove IRs in the protoplasmic membrane, and insulin also affects Aβ accumulation and systemic tau phosphorylation (Zhao et al., 2008).

The insulin-degrading enzyme (IDE) is also known to degrade other substrates such as Aβ (Farris et al., 2003). When insulin levels increase, IDE expression is activated, and it inhibits long-term insulin activity. However, in an insulin-resistant state, because IDE is used to remove insulin, senile plaques are formed in which IDE cannot lower Aβ (Shiiki et al., 2004).

GSK3-β, which is the most widely studied tau kinase, is also involved in Aβ production (Jeon et al., 2015). A study showed that GSK3-β, which is a multifunctional Ser/Thr kinase affected by tau phosphorylation and aggregation inhibition, improved learning and memory and reduced tau phosphorylation in an AD transgenic mouse model (Farr et al., 2016). In addition, GSK3-β expression was suppressed in an AD-pathology mouse model. In particular, intranasal insulin injection has been shown to help improve memory by maintaining serum insulin and glucose levels (Benedict et al., 2004), suggesting that insulin is a therapeutic target for AD.

In addition to PI3K/AKT and Ras/Raf/MAPK insulin signaling pathways, mTOR and its downstream targets that regulate neuronal survival and nutrient sensing play roles in AD pathogenesis; however, these roles are not well-defined. mTOR regulates protein synthesis by phosphorylating the key substrates of the translational machinery, namely, the eukaryotic initiation factor 4E-binding protein and p70S6 kinase. Rapamycin inhibits mTOR *in vivo* and halts cellular growth and proliferation (Showkat et al., 2014). Also, genetic inhibition of mTOR reduces the level of memory loss, improves cognitive function and reduces tan and Aβ deposits (Kaeberlein and Galvan, 2019). It is hypothesized that in an insulin-resistant state, these downstream signaling pathways are compromised, leading to increased levels of Aβ oligomers and hyperphosphorylated tau. These increased levels of Aβ oligomers and hyperphosphorylated tau occur not only due to a dysregulation of downstream kinases but also due to an impairment of autophagic clearance that arises as a result of an imbalance of the mTOR and autophagy pathways. Autophagic dysfunction which is recently gaining attention feature of AD causes the progressive accumulation of toxic proteins and eventually leads to neuronal death (Orr and Oddo, 2013).

## Linking AD and T2DM

Alzheimer’s disease, a degenerative brain disease, is the most common cause of dementia, with clinical features including gradual decline of cognitive function, amnesia, behavioral and personality changes, and pathological features including extracellular Aβ plaques and intracellular NFT intraneuronal deposition, tau protein degeneration, and severe neuronal loss in the brain tissue (Saxena, 2010). Most AD treatments have focused on Aβ but have failed to look at AD from various perspectives, for example, relating AD to obesity and T2DM (Kang et al., 2017). Considering the relationship between AD and T2DM, it might be possible to treat AD with T2DM drugs. For instance, Thiazolidinedlones (TZDs), such as pioglitazone and rosiglitazone, are PPARγ agonists used as anti-diabetic drugs, that induce a decrease in plasma free fatty acid concentration and fasting hyperglycemia through an insulin-reducing effect. A recent pioglitazone-related study found that it may be of therapeutic benefit, showing a significant reduction in Aβ and tau pathology measured in cerebral blood flow from patients with early-stage and mild to moderate AD patients (Pérez and Quintanilla, 2015).

According to the Mayo Clinic Alzheimer Disease Patient Registry, 80% of patients with AD have impaired glucose tolerance or diabetes (Janson et al., 2004). Epidemiological studies have shown that T2DM induces cognitive impairment and that T2DM patients are 1.5−2 times more likely to be diagnosed with dementia than healthy individuals are (Biessels et al., 2014). There is also evidence of cellular insulin resistance or insulin deficiency in the brains of patients with AD, including non-diabetic patients (Vijan, 2015).

Type 2 diabetes mellitus is a chronic metabolic disorder that can damage blood vessels, nerves, eyes, and kidneys and causes serious complications. Typical symptoms of T2DM associated with insulin dysfunction, including hyperglycemia, insulin resistance, and relative insulin deficiency, also induce the accumulation of Aβ in the brain, contributing to AD pathogenesis (Ramos-Rodriguez et al., 2017).

**FIGURE 1:**
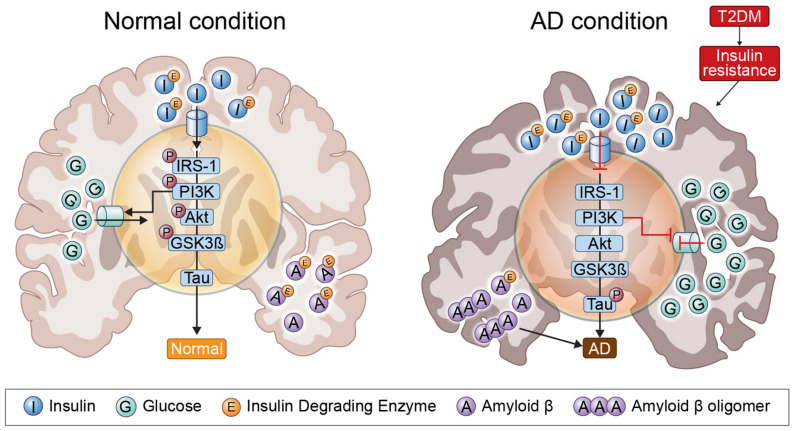
Insulin signaling in the normal condition brain and AD brain. In insulin-resistant state induced by T2DM, as insulin signaling is impaired, insulin is unable to bind to an insulin receptor, and multiple tyrosine residues are not auto-phosphorylated to activate insulin receptor substrate 1, which mediates downstream signaling through phosphoinositide 3-kinase. Therefore, GSK3-β is overactivated and tau is phosphorylated.

Several pathogenic mechanisms overlap the two diseases, including dysregulation of glucose and insulin signals, increased inflammation, Aβ deposition, mitochondrial dysfunction, and oxidative stress (Liu et al., 2011). Insulin resistance and deficiency are increased by abnormally creating insulin signaling through PI3K/Akt/GSK3-β signals; GSK3-β activation is an important component of NFT and can lead to hyperphosphorylated tau (Zheng et al., 2015). In addition, IDE associated with insulin signaling plays an important role in insulin and Aβ clearance, so that impaired IDE function can cause AD and T2DM (Ramos-Rodriguez et al., 2017). IRS1 plays an important role in transferring insulin and IGF-1 receptor signals to signal adapter proteins and the intracellular pathway. PI3K/AKT kinase pathway and IRS1 dysfunction causes AD and T2DM (illustrated in Figure 1).

## AD and T2DM Drugs

### Expressing Organs and Function of Dipeptidyl Peptidase-4

When food is ingested, a series of hormones are secreted by epithelial cells of the small intestine to increase insulin secretion. When glucose is orally administered, insulin secretion from the pancreas increases, and this phenomenon is called the incretin effect. A typical incretin is an endogenous peptide that is mainly synthesized and secreted by enteroendocrine L cells into the gastrointestinal peptide hormone (GLP-1). Physiologically, it promotes β cell proliferation, improves β cell function, decreases β cell apoptosis, increases insulin secretion, and regulates glucose homeostasis (Graaf et al., 2016; Keshava et al., 2017). Glucagon-like peptide-1 receptor (GLP-1R) is widely expressed in the hippocampus, hypothalamus, cortex, nucleus basalis of the Meynert, choroidal plexus, and nucleus of the solitary tract (Erreger et al., 2012; Yildirim Simsir et al., 2018). This factor, which is overexpressed in the hippocampus of mice, also affects neurite growth, learning, and memory (McClean et al., 2011). In addition, GLP-1 analogs and GLP-1R agonists administered peripherally or centrally reduce Aβ deposition, prevent tau and NFT protein hyperphosphorylation, and have a neuroprotective effect against rodent AD-like neurodegeneration (Hölscher, 2018). There have also been reported to be effective for maintaining synaptic plasticity and learning and memory (McClean et al., 2011; Li et al., 2012; Xiong et al., 2013; McClean and Hölscher, 2014; Candeias et al., 2015; Wang et al., 2016).

However, GLP-1 has a short duration of reaction time as it is rapidly degraded by dipeptidyl peptidase-4 (DPP-4) present in plasma and other body fluids, such as cerebrospinal fluid (Gong et al., 2014).

Dipeptidyl peptidase-4 is a type 2 transmembrane glycoprotein with various functions. Usually, the substrates of DPP-4 are peptides with a size of 80 amino acids or less, and there are more than 35 neuropeptides and chemokines, including GLP-1, GLP, neuropeptide Y, peptide YY, substance P, and stromal cell-derived factor 1, that serve as substrates for this peptide. It is found in epithelial cells, immune cells, including T lymphocytes, various cells, such as vascular endothelial cells, and almost all tissues, including kidney, liver, adrenal gland, skeletal muscle, pancreas, lung, small intestine, bone marrow, and spleen (Chen et al., 2019).

### Expressing Organs and Function of Sodium-Glucose Cotransporter 2

Usually, 180 g of glucose per day is filtered by the kidneys and reabsorbed in the proximal tubules. Glucose reabsorption occurs *via* sodium-glucose cotransporter 2 (SGLT2) present mainly in the kidney, which comprises SGLT2 located in front of the proximal tubule and sodium-glucose cotransporter 1 (SGLT1) in the latter half. In a normal blood glucose level state, SGLT2 is responsible for approximately 97% of the reabsorption of filtered glucose, whereas and SGLT1 is responsible for approximately 3%.

Glucose reabsorption begins with active transfer of Na^+^ the extracellular region by Na^+^/K^+^ ATPase in the proximal tubule. The electrochemical force generated while moving Na^+^ extracellularly causes Na^+^ and glucose to move intracellularly *via* SGLT. One Na^+^ and glucose molecule move together through SGLT2, and two Na^+^ and glucose molecules move together through SGLT1. When the glucose concentration increases by the glucose transferred into the cell, glucose is reabsorbed into the bloodstream *via* the glucose transporter based on the difference in glucose concentration between the cell and epilepsy.

Recently, there have been increasing reports of the presence of SGLTs in the mammalian central nervous system (Yu et al., 2010, 2013). The receptors for SGLT1 are expressed in CA1, CA3 (regions 1 and 3 of hippocampal cornu ammonis), and the dentate gyrus hippocampal subfields, and SGLT2 has been reported to be expressed in the hippocampus, cerebellum, and blood-hippocampal barrier endothelial cells (Poppe et al., 1997; Enerson and Drewes, 2006; Shah et al., 2012; Jurcovicova, 2014).

### Pharmacological Role of DPP-4 and SGLT2 Inhibitors

Dipeptidyl peptidase-4 inhibitors (DPP-4i_*s*_) include sitagliptin, vildagliptin, saxagliptin, linagliptin, alogliptin, and gemigliptin. As hormones that increase insulin secretion are degraded by DPP-4, DPP-4i_*s*_ can be used to suppress this hormone degradation. In other words, the principle of DPP-4 is the increase in insulin secretion following food intake and the period of insulin secretion time can be improved, and blood glucose levels can be additionally improved by suppressing glucagon secretion without inducing hypoglycemia.

The SGLT2 inhibitors (SGLT2i_*s*_) include dapagliflozin, canagliflozin, empagliflozin, ipragliflozin, tofogliflozin, luseogliflozin, and ertugliflozin. These inhibitors reduce glycated hemoglobin level by 0.3–0.9% and fasting blood glucose levels by 18–36 mg/dl, regardless of use of other drugs, and decrease body weight as well as blood pressures due to drug effects on glucosuria and natriuresis. In addition, since these have an insulin-independent hypoglycemic effect, SGLT2i_*s*_ can reduce the blood glucose level even in an environment where the insulin secretory capacity is decreased. By increasing the excretion of glucose into the urine, insulin resistance can be improved, and by improving glucose toxicity, the function of pancreatic β cells can be maintained. These are diabetic treatment agents with a low risk of hypoglycemia because they facilitate the excretion of glucose in a hyperglycemic state without affecting insulin secretion.

### Regulation of DPP-4i_*s*_ in the AD Brain

**FIGURE 2:**
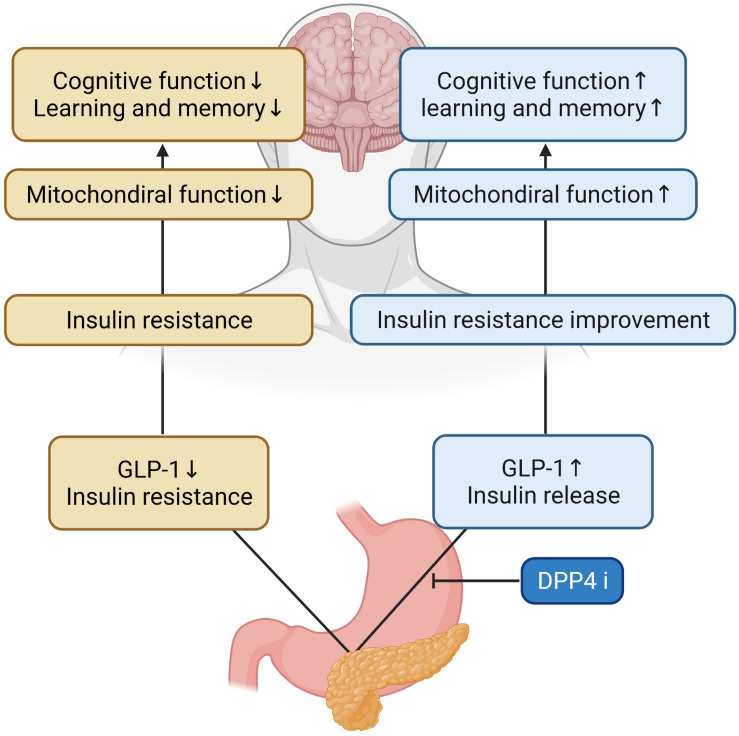
Effects of DPP-4 inhibitor in the AD brain. During DPP-4 inhibitor treatment, GLP-1 increases and insulin is secreted, which improves insulin resistance and mitochondrial function in the brain. Therefore, cognitive function and learning and memory ability are improved.

Glucagon-like peptide-1 signaling in the brain regulates glucose metabolism. Inhibitors of DPP-4 improve neuronal insulin resistance by restoring insulin-induced phosphorylation of neuronal IR, IRS1 phosphorylation, and AKT/PKB-Ser phosphorylation, resulting in the brain mitochondrial dysfunction. Previous studies on diabetes-related AD rat models have demonstrated that GLP-1 positively affects learning and memory (Chen et al., 2012). In addition, a recent study has shown that DPP4i_*s*_ can increase the levels of active GLP-1 in the brain and improve memory behaviors in AD mice models (D’Amico et al., 2010). These have also been shown to improve spatial learning and memory ability and protect synaptic proteins by increasing GLP-1 and GLP-1R expression levels in the hippocampus and cortex of AD mice (Pipatpiboon et al., 2013). Cognitive function was improved as a result of the administration of DPP-4i_*s*_ and quercetin (3,3′,4′,5,7-pentahydroxyflavone) found in vegetables and fruits which is one of the major groups of polyphenols with effects on inflammation, diabetes and the nervous system in a study (Babaei et al., 2018; Li et al., 2019), and DPP-4i_*s*_ was shown to ameliorate memory impairment, increase GLP-1 levels in the brain which acts as a neuroprotective agent (Holst et al., 2011) and could lead to improved brain and hippocampal mitochondrial function and reduced brain MDA (Pintana et al., 2013), and significantly reduce nitrosative stress, inflammation hallmarks, and Aβ deposits (D’Amico et al., 2010; Kosaraju et al., 2013a, 2017). These inhibitors showed a time-dependent improvement in memory retention and dose-dependent attenuation of Aβ, tau phosphorylation, and inflammatory markers, and AD-associated proteins were decreased in the hippocampus following DPP-4i_*s*_ administration (Kosaraju et al., 2013b; Ma et al., 2018). The combination of DPP-4i and memantine could reduce the expression of APP and phosphorylated tau protein (Khalaf et al., 2019). Inhibitors of DPP-4 alleviated cognitive deficits in 3xTG AD mice. These improve incretin levels in the brain and reduce Aβ deposition, tau phosphorylation, and neuroinflammation (Thomas et al., 2008) and can significantly protect against Aβ-induced cytotoxicity, and inhibit the activation of GSK3-β and tau hyperphosphorylation by restoring downstream insulin signaling. Inhibitors of DPP-4 ameliorated Aβ-induced mitochondrial dysfunction and intracellular reactive oxygen species (ROS) generation and upregulated *Sirt1* expression (Kosaraju et al., 2017). HFD rats had brain mitochondrial dysfunction as shown by increased ROS production, mitochondrial depolarization, and mitochondrial swelling. In the mitochondria, it has been shown that increased levels of ROS could cause the opening of the inner membrane anion channel (IMAC), thus leading to mitochondrial membrane depolarization (Zorov et al., 2006). The depolarization of mitochondria could also lead to the dysfunction of mitochondria to produce ATP synthesis (Aon et al., 2006). Furthermore, increased ROS levels could play a role in the cognitive decline observed in HFD rats (Table 1 for a summary, illustrated in Figure 2).

**TABLE 1:** Research of DPP-4 inhibitors in AD models.

Drug name	Drug type	Studies in AD models
Dipeptidyl peptidase-4 Inhibitors	Sitagliptin Vildagliptin Saxagliptin Linagliptin Alogliptin Gemigliptin	DPP-4 i improved spatial learning and memory ability and protected synaptic proteins by increasing GLP-1 and GLP-1R expression levels in the hippocampus and cortex of the brain in AD mice
		Cognitive function was improved as a result of administration of DPP-4 i and quercetin
		DPP-4 i ameliorated memory impairment, increased GLP-1 level in the brain, significantly reduced nitrosative stress, inflammation hallmarks, and Aβ deposits
		DPP-4 i significantly protected against Aβ-induced cytotoxicity, and inhibited the activation of GSK3-β and tau hyperphosphorylation by restoring insulin downstream signaling. DPP-4 i ameliorated Aβ-induced mitochondrial dysfunction and intracellular ROS generation, and upregulated *Sirt1* expression
		DPP-4 i showed a time-dependent improvement in memory retention and AD-associated proteins such as tau phosphorylation were decreased in the hippocampus with DPP-4 i administration
		The combination of DPP-4i and memantine could reduce the expression of APP, and phosphorylated tau protein
		DPP-4 i could alleviate cognitive deficits in 3xTG AD mice. It improved incretin levels in the brain and reduced Aβ, tau phosphorylation, and neuroinflammation

### Regulation of SGLT2i_*s*_ in the AD Brain

**FIGURE 3:**
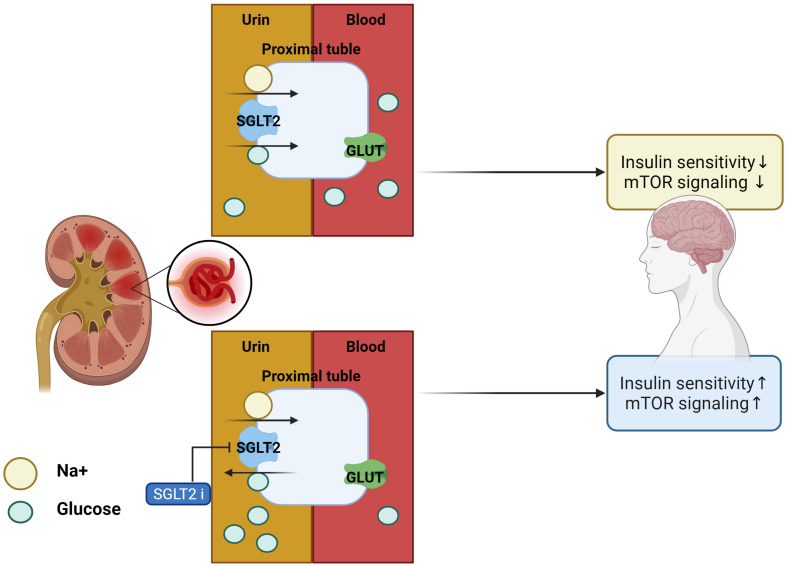
Effects of SGLT2 inhibitor in the AD brain. With SGLT2 inhibitor, insulin sensitivity and mTOR signaling in the brain are improved, as excess glucose from insulin resistance is filtered out by the kidneys.

Inhibitors of SGLT2 not only improve peripheral insulin sensitivity and reduce body weight (Xu et al., 2017) but also improve brain mitochondrial function and insulin signaling, and reduce cell death. Furthermore, SGLT2i_*s*_ prevent cognitive decline and protect synaptic plasticity in the hippocampus (Sa-Nguanmoo et al., 2017). Inhibitors of SGLT2 reduced the accumulation of Aβ in the cortical region of AD-T2DM mice (APP/PS1xdb/db mice) which is a genetically diabetic model of T2DM and showed the same effect on the amount of tau induced pathological cerebral atrophy (Wiciński et al., 2020). SGLT2i_*s*_-mediated mTOR inhibition, through continuous loss of glucose in the urine, routinely restores a reliable, overnight catabolic-fasted state in older, inactive individuals and re-establishes the benefits associated wit circadian catabolic/anabolic metabolism (e.g., reactivation of the endo-lysosomal pathway through inhibition of mTOR), removal and replacement of dysfunctional organelles/proteins, and lowering of blood pressure through mTOR-mediated modulation of sympathetic tone. Unrestrained chronic mTOR activation may be responsible for sustaining metabolic and mitochondrial dysfunction in AD, driving the breakdown of the BBB *via* endothelial cell dysfunction, as well as driving the hyperphosphorylation of tau, and formation of amyloid plaques in the brain (Mueed et al., 2018). These inhibitors can restore mTOR signaling through mTOR inhibition and prevent the progression of AD pathology (Esterline et al., 2020). In addition, SGLT2i_*s*_ physiologically elevates blood ketone bodies such as β-hydroxybutyrate (Kim et al., 2019), which can modulate NLRP3 inflammasome-IL-1β signaling (Kim et al., 2020), a key pathologic pathway in AD (Heneka et al., 2013). Decreased blood glucose levels were seen in db/db mice after 10 weeks of treatment with SGLT2i_*s*_ for T2DM. These inhibitors not only ameliorated albuminuria and glomerular injury in db/db mice but also significantly prevented the impairment of cognitive function, which was associated with the attenuation of cerebral oxidative stress and increase in cerebral brain-derived neurotrophic factor level (Lin et al., 2014). SGLT2i_*s*_ treatment significantly attenuated cerebral oxidative stress and DNA oxidative damage in db/db mice, as shown by the reduction of cerebral superoxide and 8-OHdG, and this attenuation of cerebral oxidative stress was associated with the reduction of cerebral NADPH oxidase subunit. Therefore, the improvement of cognitive function by SGLT2i_*s*_ seems to be attributed to the attenuation of oxidative stress. Moreover, the effect of SGLT2i_*s*_ treatment on cerebral BDNF, since BDNF, a key protein promoting memory and survival of neurons, is significantly reduced in diabetic patients, and diabetic animals including db/db mice and the decrease in cerebral BDNF is shown to be associated with cognitive decline (Lin et al., 2014; Table 2 for a summary, illustrated in Figure 3).

**TABLE 2:** Research of SGLT2 inhibitors in AD models.

Drug name	Drug type	Studies in AD models
Sodium-glucose cotransporter 2 inhibitors	Dapagliflozin Canagliflozin Empagliflozin Ipragliflozin Tofogliflozin Luseogliflozin Ertugliflozin	SGLT2 i not only improved peripheral insulin sensitivity and reduced increasing of body weight, but also improved brain mitochondrial function, insulin signaling, and reduction of cell death
		SGLT2 i prevented cognitive decline and protect synaptic plasticity in the hippocampus
		SGLT2 i reduced the accumulation of Aβ in the cortical region of Aβ precursor protein (APP)/PS1xdb/db mice and showed the same effect on the amount of tau pathological cerebral atrophy
		SGLT2 i restored mTOR signaling through mTOR inhibition and prevented the progression of the pathology of AD
		SGLT2 i physiologically elevates blood ketone bodies such as β-hydroxybutyrate, which can modulate NLRP3 inflammasome-IL-1β signaling, and a key pathologic pathway in AD
		SGLT2 i not only ameliorated albuminuria, and glomerular injury in db/db mice but also significantly prevented the impairment of cognitive function in db/db mice, which was associated with the attenuation of cerebral oxidative stress, and the increase in cerebral brain-derived neurotrophic factor
		SGLT2 i seems to be attributed to the attenuation of oxidative stress and since BDNF, the effect of SGLT2 i treatment promotes memory, and survival of neurons

### Clinical Evidence in Therapeutic Effects of DPP-4i and SGLT2i on Dementia

Majority of reports regarding the effects of anti-diabetic agents on dementia have been investigated from retrospective studies. In 240 elderly patients with T2DM affected by mild cognitive impairment (MCI), 2 years treatment group of DPP-4i significantly improve cognitive functions measured by mini-mental state examination (MMSE), compared to the sulfonylurea which increases endogenous release of insulin from pancreatic β cells group (Rizzo et al., 2014). A prospective, non-randomized study showed that sitagliptin therapy prevented from the decline of MMSE during 6 months in old T2DM (Isik et al., 2017). Using a health insurance claim database in Korea, DPP-4i use demonstrated a significant 46% decrease in AD development among elderly T2DM (Kim et al., 2018). Although there are few data on SGLT2i, one randomized clinical trial reported no changes in MMSE after 12-month treatment of incretins vs. SGLT2i (Perna et al., 2018). Further larger and well-designed clinical studies are needed to evaluate the neuroprotective effects of DPP-4i and SGLT2i.

## Conclusion

Recent studies have identified the key mechanisms by which the brain becomes resistant to insulin in AD and how impaired insulin signaling in AD is linked to memory impairment (Vieira et al., 2018). In this review, we describe the connections between AD and T2DM. Although the reason why many T2DM patients develop AD is not clear, the two diseases are associated with insulin resistance. Significant effort is required to identify the common pathological and molecular mechanisms between AD and T2DM, which will help better understand the onset and development of both diseases. Therefore, novel approaches to identify biomarkers for detecting early-stage of AD will likely increase the efficacy of anti-diabetic agents and allow treatment before severe neuronal dysfunction occurs in the AD brains.

## Author Contributions

AYS and SB wrote the manuscript. AYS, SB, JYK, Y-HL, and JEL participated in the discussion and revision. JEL designed and edited the final manuscript. All authors read and approved the final manuscript.

## Conflict of Interest

The authors declare that the research was conducted in the absence of any commercial or financial relationships that could be construed as a potential conflict of interest.

## Publisher’s Note

All claims expressed in this article are solely those of the authors and do not necessarily represent those of their affiliated organizations, or those of the publisher, the editors and the reviewers. Any product that may be evaluated in this article, or claim that may be made by its manufacturer, is not guaranteed or endorsed by the publisher.
